# Factors Driving the Popularity and Virality of COVID-19 Vaccine Discourse on Twitter: Text Mining and Data Visualization Study

**DOI:** 10.2196/32814

**Published:** 2021-12-03

**Authors:** Jueman Zhang, Yi Wang, Molu Shi, Xiuli Wang

**Affiliations:** 1 Polk School of Communications Long Island University Brooklyn, NY United States; 2 Department of Communication University of Louisville Louisville, KY United States; 3 Louisville, KY United States; 4 School of New Media Peking University Beijing China

**Keywords:** COVID-19, vaccine, topic modeling, LDA, valence, share, viral, Twitter, social media

## Abstract

**Background:**

COVID-19 vaccination is considered a critical prevention measure to help end the pandemic. Social media platforms such as Twitter have played an important role in the public discussion about COVID-19 vaccines.

**Objective:**

The aim of this study was to investigate message-level drivers of the popularity and virality of tweets about COVID-19 vaccines using machine-based text-mining techniques. We further aimed to examine the topic communities of the most liked and most retweeted tweets using network analysis and visualization.

**Methods:**

We collected US-based English-language public tweets about COVID-19 vaccines from January 1, 2020, to April 30, 2021 (N=501,531). Topic modeling and sentiment analysis were used to identify latent topics and valence, which together with autoextracted information about media presence, linguistic features, and account verification were used in regression models to predict likes and retweets. Among the 2500 most liked tweets and 2500 most retweeted tweets, network analysis and visualization were used to detect topic communities and present the relationship between the topics and the tweets.

**Results:**

Topic modeling yielded 12 topics. The regression analyses showed that 8 topics positively predicted likes and 7 topics positively predicted retweets, among which the topic of vaccine development and people’s views and that of vaccine efficacy and rollout had relatively larger effects. Network analysis and visualization revealed that the 2500 most liked and most retweeted retweets clustered around the topics of vaccine access, vaccine efficacy and rollout, vaccine development and people’s views, and vaccination status. The overall valence of the tweets was positive. Positive valence increased likes, but valence did not affect retweets. Media (photo, video, gif) presence and account verification increased likes and retweets. Linguistic features had mixed effects on likes and retweets.

**Conclusions:**

This study suggests the public interest in and demand for information about vaccine development and people’s views, and about vaccine efficacy and rollout. These topics, along with the use of media and verified accounts, have enhanced the popularity and virality of tweets. These topics could be addressed in vaccine campaigns to help the diffusion of content on Twitter.

## Introduction

### Background

Since the World Health Organization (WHO) declared the COVID-19 outbreak a pandemic in March 2020 [1], the United States has seen the highest number of confirmed cases and deaths [2]. Many health organizations, including the WHO [3] and the US Centers for Disease Control and Prevention (CDC) [4], consider vaccination as a critical prevention measure to help end the pandemic and restore society to its normal status. Owing to remarkable advances in vaccinology, scientists developed COVID-19 vaccines within an unprecedented short time. In December 2021, less than 1 year after the virus was identified, the first two vaccines were approved for emergency use in the United States: the Pfizer-BioNTech vaccine and the Moderna vaccine [5]. Both of these vaccines use messenger RNA (mRNA)-based technology, which had not been approved previously for general use in humans [5]. Johnson & Johnson’s Janssen vaccine, which is based on a slightly more mature technology of a viral vector, became the third vaccine approved for emergency use in the United States in February 2020 [6]. Owing to their novelty, COVID-19 vaccines had potential to fuel the existing vaccine debate, including arguments over vaccine safety and effectiveness, which had received notable attention in recent years before the pandemic [7]. In addition, political polarization, reaffirmed in the 2020 presidential election, was manifested in a wide range of issues, including responses to the COVID-19 pandemic [8] and vaccines [9]. Generally, Democrats had more favorable attitudes toward COVID-19 vaccines than Republicans [9]. These political fissures further had potential to propel the vaccine debate. Amidst the heated discussion of COVID-19 vaccines, the United States has been rolling out the most massive vaccination campaign in its history to fight against the pandemic [10].

Investigating public discourse about COVID-19 vaccines will shed light on people’s perception and attitudes. As a major social media platform and a vital source for text-based public discourse, Twitter has been studied to understand public discourse about vaccines in general [11-14] and about specific vaccines, including COVID-19 vaccines [15,16]. Text-mining techniques have been increasingly used in recent research to investigate tweets about the COVID-19 pandemic (eg, [17-21]) and about COVID-19 vaccines [15,16]. These studies have employed machine learning algorithms to automatically analyze massive amounts of tweets and capture latent textual information such as topics, sentiment, and trends.

Although text mining is clearly an effective way to identify underlying textual clusters and patterns from vast amounts of tweets, less is known about how such information can help to understand the diffusion of information and opinions on Twitter. The aim of this study was to investigate message-level drivers of the popularity and virality of tweets about COVID-19 vaccines using text-mining techniques. Specifically, the objective of the study was to investigate how text-mined topics and valence, together with social media message features affect likes and retweets. Another aim of the study was to examine the topic communities of the most liked and most retweeted tweets using network analysis and visualization. These findings have implications for the direction of vaccine campaigns.

### Literature Review

The extent to which a message results in optimal diffusion on social media can be assessed by users’ favorable responses such as clicking “like” and “share” buttons to overtly indicate individual interest and support [22,23]. On Twitter, users can click on the “Like” icon to show appreciation for a tweet or on the “Retweet” icon to share it publicly with their followers [24]. Prior research has considered the like count of a tweet as an indicator of its popularity and the retweet count of a tweet as an indicator of its virality [23,25]. Drawing on these studies [23,25], we assessed the popularity of a tweet by the number of likes and assessed the virality of a tweet by the number of retweets. Compared with liking, retweeting is a more social behavior [26]. For both responses, the bandwagon effect postulates that the adoption of trends increases more with respect to the number of people who have already done so [22].

This study investigated three categories of message-level factors that, according to prior research, can drive the diffusion of media content online: information, emotion, and social media message features. As Twitter is a major source of text-based information, we drew on the literature related to the social transmission of online text information, including news articles and tweets. Past research on the virality of online news has suggested two categories of determinants: informational and emotional. From the informational perspective, information utility, as gauged by overall content usefulness, was found to prompt social media sharing of general news articles [27]. In the health context, a content attribute that taps into information utility is the presence of efficacy information [26], which provides ways to promote health or overcome a health risk [28]. Research has shown that overall content usefulness and presence of efficacy information both facilitate viewing and sharing of health news articles on social media [26]. In the situation of the COVID-19 pandemic, gaps in knowledge about the new coronavirus was evident in the United States early on [29] and demand for information of practical value was expected [25,30]. In addition, according to the uncertainty reduction theory, to alleviate risks in crises, people intend to engage in uncertainty reduction efforts by collecting credible information and sharing with others [25]. Nanath and Joy’s [25] text mining study revealed that the optimism and solution topic as well as the mental health topic were positive predictors of retweet counts of COVID-19–related tweets. In addition to information utility, novel content in health news has been found to increase sharing [26]. COVID-19 vaccines were newly developed to help fight off the new coronavirus; thus, content related to aspects such as development and efficacy had the intrinsic feature of novelty and could potentially help to close the knowledge gaps.

Past research has generally shown that there were more positive than negative tweets on Twitter about vaccines in general [11-13] and about COVID-19 vaccines in particular [15,16]. Although positive content has been found to increase likes on social media [22,23], the findings are mixed regarding the impact of valence on the virality of online content. Berger and Milkman [27] found that positive sentiment increased social media sharing of general news. A plausible explanation is that positive sharing reflects the positivity of the sender [26], which may enhance self-presentation [31] and identity communication [27]. However, Nanath and Joy [25] found that negative emotions increased the social transmission of COVID-19–related tweets. Moreover, Blankenship et al [11] revealed that antivaccine tweets were retweeted more than provaccine tweets. In comparison, Kim [26] revealed that content valence was unrelated to the virality of health news on social media.

In addition to content topic and valence, social media message features, including media presence, linguistic features, and account verification, could impact the popularity and virality of online content. Media presence and linguistic features can affect content processing fluency and further affect favorable online responses such as liking and retweeting. Content on social media may be of any mode such as text, photos, and videos. Past research has shown that a tweet with embedded media (ie, a photo or a video) stimulates likes and retweets [23]. It is postulated that the cognitive processing of photos is more fluent than that of words as it is faster to activate the semantic meaning of photos than that of words [32,33]. Therefore, tweets featuring embedded media are more likely to trigger favorable online responses.

In comparison, past research has revealed that linguistic features such as the number of hashtags, mentions, and external links decrease likes [23] and retweets [23,25]. It is suggested that these features increase content processing disfluency in two aspects. First, compared to the black color adopted by text, the blue color adopted by hashtags, mentions, and external links decreases the font-background contrast and causes visual perpetual disfluency [23,34]. Second, the nonalphanumeric symbols used by hashtags, mentions, and external links (ie, #, @, ://) create orthographical disfluency [23,35]. The content disfluency requires more cognitive effort to process the message and hence decreases favorable responses [23].

Finally, account features could potentially affect likes and retweets. In the face of information explosion in the digital age, account authenticity could be of particular importance in the diffusion of information. On Twitter, verified accounts have a blue badge next to the profile name to let users know that it is authentic. Twitter paused public submissions for account verification in 2017 and reopened the gate using a new application process in May 2021 [36]. The end date of our data retrieval was April 30, 2021, and therefore the data did not reflect the newly verified accounts. In addition, it is noteworthy that the tweets posted by verified accounts may not be verified.

### Research Model and Questions

This study contributes to the literature by providing a conceptual model to understand the combined effects of the three above-mentioned categories of factors—content topics, content valance, and social media message features, including media presence, linguistic features, and account verification—on the popularity and virality of tweets about COVID-19 vaccines. We employed topic modeling to identify latent topics of tweets. We employed sentiment analysis to assess the valence of tweets. Automated extraction generated data about social media features. Therefore, we put forward the following research questions:

Research question 1 (RQ1): How do content topics, content valence, and social media message features affect the popularity of tweets about COVID-19 vaccines?

Research question 2 (RQ2): How do content topics, content valence, and social media message features affect the virality of tweets about COVID-19 vaccines?

In addition, among the 2500 most liked and most retweeted tweets, respectively, we used network analysis and visualization to detect topic communities and present the relationship between the topics and the tweets. We had the following research questions:

Research question 3 (RQ3): What are the salient topics of the most liked tweets?

Research question 4 (RQ4): What are the salient topics of the most retweeted tweets?

This study can help to advance knowledge on complex drivers of the popularity and virality of tweets about COVID-19 vaccines using machine-based text mining and network visualization in the context of a heated vaccine debate in the United States. These findings offer practical implications for health practitioners to employ more effective social media content.

## Methods

### Data Source

We collected publicly available original tweets about COVID-19 vaccines from January 1, 2020, to April 30, 2021, using snscrape [37], which were further filtered according to user profile data to include only English-language tweets and those from US-based users. This approach resulted in 501,531 tweets recorded in the final dataset.

Drawing on prior social media studies on vaccines [38,39], we developed keywords by balancing the general COVID-19 vaccine information with brand-specific information. As of April 30, 2021, which was our data retrieval end date, Pfizer-BioNTech, Moderna, and Johnson & Johnson/Janssen vaccines were authorized for emergency use in the United States [40]. At that time, the three vaccines, together with the AstraZeneca vaccine, had conditional marketing authorizations in European Union countries [41]. Although the AstraZeneca vaccine was not used in the United States, it garnered media and public attention in the United States, and therefore we also included this brand in the search. In addition, as COVID-19 vaccines varied in terms of the underlying technology, we considered technology-specific information. Pfizer-BioNTech and Moderna used mRNA technology, and Johnson & Johnson and AstraZeneca-Oxford used viral vector technology. Moreover, we checked government Twitter accounts such as the US CDC and Food and Drug Administration accounts to explore hashtags. Finally, the following strategy was used to scrape Twitter data. A tweet had to contain the keyword (case-insensitive unless otherwise specified) “vaccine,” together with one of the keywords “COVID,” “COVID19,” “COVID-19,” “Pfizer,” “Pfizer-BioNTech,” “Moderna,” “Johnson & Johnson,” “Janssen,” “AstraZeneca,” and “Oxford-AstraZeneca”; or contain the keyword “vaccine” together with one of the following combinations: “mRNA” and “COVID,” “viral vector” and “COVID,” and “adenovirus” and “COVID”; or contain either of the two hashtags “#covid19vaccine” and “#covidvaccine.”

### Data Processing

The final dataset was preprocessed via genism [42] for topic modeling and sentiment analysis. We tokenized each tweet as a list of words [43], and removed high-frequency stop words such as “https” and “covid,” in addition to the standard nltk stop words library [44], which were not expected to contribute to the uniqueness of each topic. The text corpus was then trained to recognize frequent bigrams such as “New York,” using a gensim bigram model [42]. Next, all words were lemmatized to their dictionary form [43] to reduce redundancy in the bag of words (BOW) encoding. Finally, these lemmatized single words (ie, unigrams) and bigrams recognized by the bigram model were used to build the BOW representation for our latent Dirichlet allocation (LDA) model. That is, the corpus was encoded as a vector space, with each vector component representing a lemma.

### Measures

#### Like Count

The like count of each tweet, which is the number of likes a tweet gets, was captured in the data set. As a small number of tweets generated a great number of likes, the distribution was right-skewed. To reduce right skewness, we used the natural logarithm of like counts in statistical analyses, as in past research [23].

#### Retweet Count

The retweet count of each tweet, which is the number of retweets a tweet gets, was captured in the data set. Similar to like counts, retweet counts had a right-skewed distribution. To reduce right skewness, we used the natural logarithm of retweet counts in statistical analyses, as in past research [23,25].

#### Content Topic

The tweets were subjected to topic modeling using the LDA model [45]. Topic modeling is a commonly used unsupervised learning method that generates a probabilistic model for the corpus of text data [46]. As one of the two main topic models [46], LDA is increasingly being used to analyze textual data [47], including tweets (eg, [16-18,20,25]).

LDA depends on two matrices to define the latent topical structure: the word-topic matrix and the document-topic matrix [47]. In our study, a document was a tweet. The general idea is that a tweet is represented by a Dirichlet distribution of latent topics, where each latent topic is represented by a Dirichlet distribution of words [46].

The word-topic matrix reveals the conditional probability with which a word is likely to occur in a topic. The word-topic matrix is used to interpret the topics. A topic can be interpreted by examining a list of the most probable words ranked solely by their frequency to occur in that topic, using 3 to 30 words [48]. To aid topic interpretation, we also considered the ranking of the most probable topic-specific words by both frequency and relevance, as suggested by Sievert and Shirley [48]. The relevance for ranking words within a topic is indexed by a weight parameter, λ, with a value ranging from 0 to 1. A value closer to 0 highlights rare but exclusive words for the topic and a value closer to 1 highlights frequent but not necessarily exclusive words for the topic [48]. We adopted the recommended λ of 0.6 [48]. Lastly, we reviewed sample tweets with the highest topic-specific loadings to finalize topic interpretations.

The document-topic matrix reveals the conditional probability with which a topic is likely to occur in a tweet. In other words, it reveals the topic loadings for each tweet. The information was used in the regression models for prediction as well as in network analysis and visualization. The topic loading value ranges from 0 to 1, with a value closer to 1 indicating the higher topic loading of a tweet.

#### Content Valence

We used TextBlob [49], an open-source python library, to generate the valence score of each tweet. The range of the valence score is from –1 to 1, with the value of –1 indicating the most negative and the value of 1 indicating the most positive valence.

#### Media Presence

Data on whether a tweet had a photo, gif, or video were extracted, respectively.

#### Linguistic Features

The numbers of hashtags, mentions, and hyperlinks were extracted, respectively.

#### Account Verification

For each tweet, whether the account that posted it was verified or not was extracted.

### Data Analysis

We performed linear regression analyses to examine the predictors of likes and retweets. Since the purpose of the study was to investigate the factors that affected the popularity and virality of tweets as indexed by like counts and retweet counts, we only considered the tweets that were liked and retweeted, as in past research [23,25]. In the models, the log-transformed like counts and retweet counts were respectively regressed on 12 topic loadings extracted from topic modeling, the valence score generated from sentiment analysis, three variables of media presence, three variables of linguistic features, and account verification.

### Network Analysis and Visualization

We used two-mode visualization to present the relationship between topics and the 2500 most liked tweets and the 2500 most retweeted tweets, respectively. To prepare data for rendering each relationship network, we created a node list consisting of topic and tweet nodes, and an edge list consisting of tweet IDs, the topics each tweet was connected to, and an edge weight representing the topic loading of each tweet. Each topic node with its name was sized in proportion to the sum of topic loadings of all tweets. To assist the viewer in discerning topics, we used a community detection algorithm built in Gephi [50], which is based on the Louvain modularity method that has been used in prior research [12]. Community detection algorithms [51] identify cohesive groups in the network [52,53]. In the network visualization, node color reflected topic community membership.

## Results

### Content Topics

We trained a topic model using LDA, with a search space on topic numbers from 3 to 21. Using a uniform search grid on Dirichlet concentration parameters, the model parameters were trained to optimize the coherence score *C_v_* [54], which is a likelihood measure of word cooccurrence in the same topics. The best model was achieved at 12 topics with *C_v_*=0.42. Table 1 summarizes the 12 topics. Interpretation of each topic was based on the top 10 probable words ranked solely by frequency and jointly by frequency and relevance, as well as review of sample tweets with high topic-specific loadings.

**Table 1:** Summary of topics and valence.

Topic number	Topic label	Top 10 words by frequency (λ=1)	Top 10 words by frequency and relevance (λ=0.6)	Valence
1	Vaccine access	vaccine, community, health, help, access, need, work, pandemic, country, support	vaccine, community, health, access, help, support, effort, global, distribution, ensure	0.137
2	Vaccine efficacy and rollout	vaccine, case, new, variant, show, death, test, risk, virus, report	case, vaccine, variant, show, new, test, death, study, pause, report	0.147
3	Vaccine development and people’s views	vaccine, people, take, say, would, do, want, think, give, woman	vaccine, would, take, woman, people, think, enough, do, say, try	0.158
4	Vaccination status	get, vaccine, vaccinate, shot, people, shoot, vaccinated, first, fully, wait	get, vaccinate, shot, shoot, people, vaccinated, fully, family, wait, die	0.143
5	Feeling and side effect	get, vaccine, feel, go, good, day, side effect, make, work, arm	feel, get, side effect, good, go, arm, day, fact, science, normal	0.117
6	Vaccine appointment	vaccine, appointment, today, site, schedule, open, visit, call, clinic, vaccination	appointment, site, vaccine, open, schedule, visit, clinic, join, register, call	0.133
7	Vaccine availability	vaccine, available, week, say, year, question, old, last, next, come	available, question, old, year, week, say, last, next, answer, month	0.149
8	Vaccination eligibility and administration	dose, vaccine, receive, today, first, second, eligible, administer, day, start	dose, receive, second, eligible, today, first, administer, vaccine, day, begin	0.354
9	Age and issues	age, vaccine, offer, people, group, encourage, read, rollout, issue, concern	age, offer, group, encourage, rollout, reason, article, issue, explain, doctor	0.107
10	Preventive measures	safe, mask, keep, spread, stop, stay, wear, still, continue, passport	safe, mask, keep, spread, stop, stay, wear, passport, place, home	0.089
11	Student and county	retweet, check, student, event, walk, turn, county, staff, please, team	retweet, check, student, event, walk, turn, county, staff, please, team	0.093
12	Trust and communication	share, trust, watch, video, speak, play, minute, fall, head, availability	share, trust, video, speak, play, minute, watch, fall, head, availability	0.089

### Content Valence

The overall valence was positive, with a score of 0.145. The range of the valence score is from –1 to 1, with –1 indicating the most negative and 1 indicating the most positive valence. As shown in Table 1, all 12 topics were associated with positive valence.

### Determinants of Like Counts

Table 2 reveals the effects of the four categories of independent variables on the log-transformed like counts. The regression model was significant at *P*<.001 (adjusted *R^2^*=0.151). RQ1 was related to the determinants of likes. Out of the 12 latent topics identified by topic modeling, Topics 1 to 8 had weak but significant effects on likes. The valence also had a weak but significant effect on likes. Positive tweets increased likes. Media (photo, gif, or video) presence increased likes. Among linguistic features, the number of hashtags and that of external links decreased likes, whereas the number of mentions increased likes. Account verification increased likes.

**Table 2:** Linear regression models on predictors of popularity and virality of tweets.

Variables			Ln (like count)^a^ (n=286,657)				Ln (retweet count)^a^ (n=168,961)		
			*β*		*P* value		*β*		*P* value
**Topics**									
	T1: Vaccine access	.029		.048		.062		<.001	
	T2: Vaccine efficacy and rollout	.049		<.001		.077		<.001	
	T3: Vaccine development and people’s views	.055		<.001		.078		<.001	
	T4: Vaccination status	.048		<.001		.068		<.001	
	T5: Feeling and side effect	.040		<.001		.052		<.001	
	T6: Vaccine appointment	.027		<.001		.033		<.001	
	T7: Vaccine availability	.018		<.001		.019		<.001	
	T8: Vaccination eligibility	.011		<.001		.006		.08	
	T9: Age and issues	.009		.13		.009		.10	
	T10: Preventive measures	–.030		.26		–.037		.25	
	T11: Student and county	.076		.14		–.080		.14	
	T12: Trust and communication	–.079		.11		–.072		.21	
Emotion (valence)			.059		<.001		.0003		.93
**Media presence**									
	Has photo	.188		<.001		.088		<.001	
	Has gif	.019		<.001		.001		.64	
	Has video	.100		<.001		.084		<.001	
**Linguistic features**									
	Number of hashtags	–.072		<.001		–.059		<.001	
	Number of mentions	.007		.005		–.002		.45	
	Number of external links	–.126		<.001		.003		.18	
Verified account			.452		<.001		.378		<.001

^a^To account for the right skewness of the data distribution, the natural log–transformed like counts and retweet counts were used in the analyses.

### Determinants of Retweet Counts

Table 2 also reveals the effects of the four categories of independent variables on the log-transformed retweet counts. The regression model was significant at *P*<.001 (adjusted *R^2^*=0.130). RQ2 focused on the determinants of retweets. Out of the 12 latent topics identified by topic modeling, Topics 1 to 7 had weak but significant effects on retweets. The valence had no effect on retweets. Media presence of a photo or video increased retweets. Among linguistic features, the number of hashtags decreased retweets. Account verification increased retweets.

### Topic and Tweet Relationship Networks

**Figure 1:**
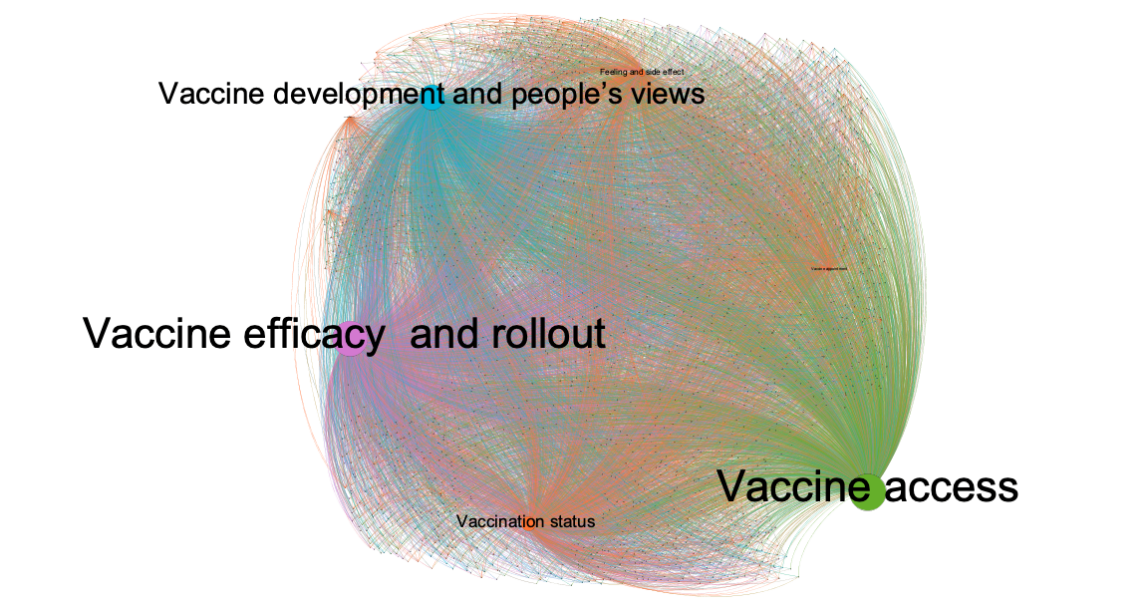
Topic communities of the 2500 most liked tweets. Two-mode visualization was used to present the relationship between topics and the 2500 most liked tweets. The topics and the tweets are connected by edges weighted by topic loadings of each tweet. Each topic node with its name is sized in proportion to the sum of topic loadings of all tweets. Colors indicate topic communities as partitioned by the Louvain algorithm.

RQ3 focused on salient topics among the most liked tweets. As shown in Figure 1, among the 2500 most liked tweets, Louvain clustering identified 4 out of the 12 topics. The tweets were clustered around vaccine access (Topic 1), followed closely by vaccine efficacy and rollout (Topic 2) and then vaccine development and people’s views (Topic 3). The other topics were not salient and presented as one remaining cluster. Each topic community was represented by one color.

Table 3 summarizes the top 10 liked paraphrased tweets, like counts, dominant topics, and topic loadings. The first most liked tweet, which was posted in July 2020 and had 91,163 likes as of April 30, 2021, was clustered around vaccine access (Topic 1). It called for Medicare for All along with free COVID testing, treatment, and vaccines.

**Figure 2:**
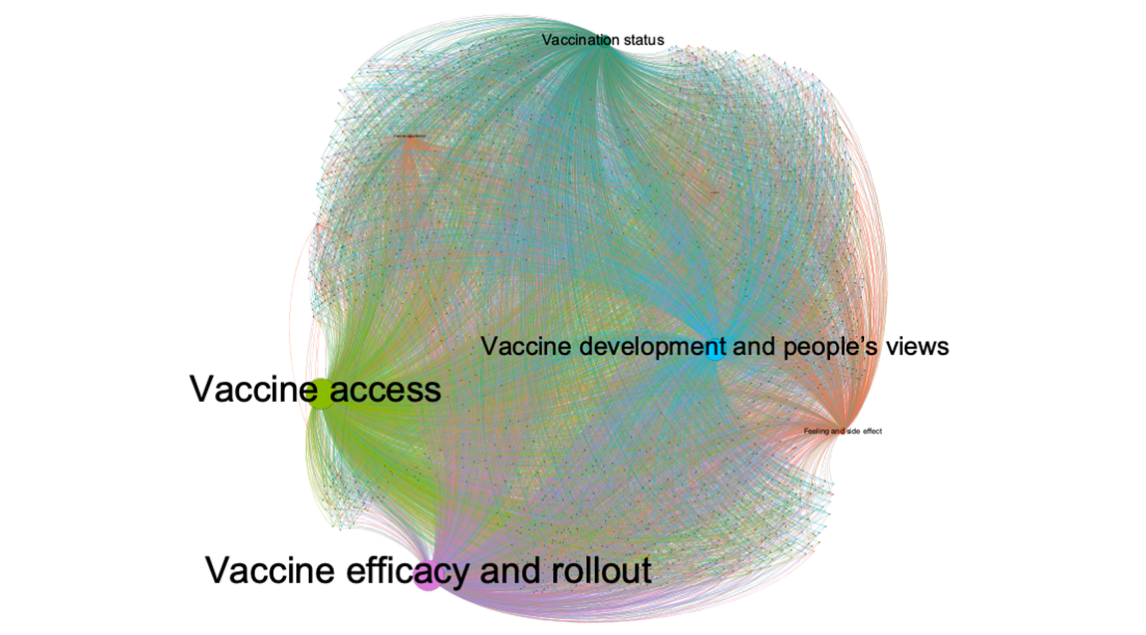
Topic communities of the 2500 most retweeted tweets. Two-mode visualization was used to present the relationship between topics and the 2500 most retweeted tweets. The topics and the tweets are connected by edges weighted by topic loadings of each tweet. Each topic node with its name is sized in proportion to the sum of topic loadings of all tweets. Colors indicate topic communities as partitioned by the Louvain algorithm.

RQ4 focused on salient topics of the most retweeted tweets. As shown in Figure 2, among the top 2500 most retweeted tweets, Louvain clustering identified 5 out of the 12 topics the LDA identified in the total tweets. The top retweeted tweets mostly clustered around vaccine efficacy and rollout (Topic 2), closely followed by access to vaccine (Topic 1), and then vaccine development and people’s views (Topic 3) and vaccination status (Topic 5). The other topics were not salient and presented as one remaining cluster. Each topic community was represented by one color.

**Table 3:** Top 10 liked paraphrased tweets.

Like rank	Like count	Tweet	Dominant topic number and label	Dominant topic loading
1	91,163	Medicare for All along with free COVID testing, treatment, and vaccines are necessities of a decent society (July 2020).^a^	Topic 1: Vaccine access	0.518
2	90,177	Trump’s attempt to deny vaccines to New York is playing politics with people’s lives (November 2020).^a^	Topic 2: Vaccine efficacy and rollout	0.578
3	63,681	I participated in Moderna experiments to see if its vaccine and booster were safe and effective (April 2021)	Topic 3: Vaccine development and people’s views	0.373
4	55,223	President Biden took credit for the vaccine from President Trump (March 2021)^a^	Topic 1: Vaccine access	0.964
5	48,631	The number of vaccine doses administered outnumbered that of new cases at a 10-to-1 ratio (February 2021)	Topic 2: Vaccine efficacy and rollout	0.514
6	46,997	I had ended my support for Trump and started taking COVID seriously. I got vaccinated, thanks to Biden and health workers (March 2021)	Topic 4: Vaccination status	0.578
7	36,753	Like with smallpox, vaccinations along with surveillance and contact tracing are essential to COVID’s elimination (April 2020)^a^	Topic 2: Vaccine efficacy and rollout	0.547
8	36,250	Pfizer’s mRNA vaccine candidate showed initial evidence of efficacy (November 2020)^a^	Topic 3: Vaccine development and people’s views	0.844
9	35,604	President Trump delivered on his goal of having a safe and effective COVID vaccine by the end of the year (May 2020)	Topic 3: Vaccine development and people’s views	0.533
10	35,514	The current vaccination pace will take 10 years to reach herd immunity. We need to speed this up (December 2020)^a^	Topic 2: Vaccine efficacy and rollout	0.385

^a^Tweet was among the top 10 liked and concurrently one of the top 10 retweeted tweets.

Table 4 summarizes the top 10 retweeted paraphrased tweets, their retweet counts, and dominant topics. The first most retweeted tweet, which was posted in December 2020 and garnered 17,427 retweets through April 2021, clustered around vaccine efficacy and rollout (Topic 2). This emphasized the long time needed to reach herd immunity based on the vaccination pace at that time.

**Table 4:** Top 10 retweeted paraphrased tweets.

Retweet rank	Retweet count	Tweet	Dominant topic number and label	Dominant topic loading
1	17,427	The current vaccination pace will take 10 years to reach herd immunity. We need to speed this up (December 2020)^a^	Topic 2: Vaccine efficacy and rollout	0.385
2	16,288	Medicare for All along with free COVID testing, treatment, and vaccines are necessities of a decent society (July 2020)^a^	Topic 1: Vaccine access	0.518
3	15,575	Trump’s attempt to deny vaccines to New York is playing politics with people’s lives (November 2020)^a^	Topic 2: Vaccine efficacy and rollout	0.578
4	14,536	The FDA^b^ and CDC^c^ recommend a pause in the use of the Johnson & Johnson COVID19 vaccine (April 2021)	Topic 1: Vaccine access	0.417
5	12,473	Pfizer’s mRNA vaccine candidate showed initial evidence of efficacy (November 2020)^a^	Topic 3: Vaccine development and people’s views	0.844
6	11,684	President Biden took credit for the vaccine from President Trump (March 2021)^a^	Topic 1: Vaccine access	0.964
7	11,046	Russian vaccine trial shows high efficacy (February 2021)	Topic 2: Vaccine efficacy and rollout	0.618
8	10,151	UK’s vaccine is safe and induces an immune reaction (July 2020)	Topic 2: Vaccine efficacy and rollout	0.844
9	8586	Like with smallpox, vaccinations along with surveillance and contact tracing are essential to COVID’s elimination (April 2020)^a^	Topic 2: Vaccine efficacy and rollout	0.547
10	8282	Why we need two doses of mRNA vaccines (April 2021)	Topic 1: Vaccine access	0.488

^a^Tweet was among the top 10 retweeted and concurrently one of the top 10 liked tweets.

^b^FDA: Food and Drug Administration.

^c^CDC: Centers for Disease Control and Prevention.

## Discussion

### Principal Results

This study investigated the combined effects of the three categories of message-level factors on the popularity and virality of tweets about COVID-19 vaccines using text-mining techniques. We also examined the topic communities of the most liked and most retweeted tweets using network analysis and visualization. In this section, we first discuss how text-mined topics and valence, together with autoextracted information about social media message features affected likes and retweets. We further discuss limitations and implications for the directions of vaccine campaigns.

Out of the 12 latent topics identified by topic modeling, Topics 1-8 increased likes and Topics 1-7 increased retweets. Vaccine development and people’s views (Topic 3) had the largest positive impact on likes and retweets, as reflected by *β* coefficients. The intrinsic novelty feature of COVID-19 vaccines could provide plausible explanations. The vaccines were newly developed to help fight off the new coronavirus, and two out of the four brands examined in the study used mRNA, a technology that had not been approved previously for general use in humans [5]. Therefore, information about vaccine development and technology was more popular and viral. Relatedly, 3 out of the top 10 liked tweets reflected Topic 3, two of which were about mRNA vaccines. One out of the top 10 retweeted tweets reflected Topic 3, which was about mRNA vaccines. The findings were consistent with those in past research that suggested the impact of novel content in the social transmission of health news [26].

Vaccine efficacy and rollout (Topic 2) had the second largest positive impact on likes and retweets, as indicated by *β* coefficients. Prior research revealed the impact of efficacy information on the virality of online health news [26] and in tweets about the COVID-19 pandemic [25]. This study also underscores the importance of efficacy information on the virality of tweets about COVID-19 vaccines.

The findings suggest that tweets focusing on the topic of vaccine development and people’s views, and the topic of vaccine efficacy and rollout highly meet the public’s needs for information during the COVID-19 pandemic, and therefore tend to become popular and viral on Twitter. It is plausible that these tweets provide useful and novel information that help to reduce uncertainty in a health crisis. Vaccine campaigns could provide more information about these topics to help the diffusion of information on social media.

It is notable that polarized political information such as that supporting a political party could be intertwined with different topics. Polarized political information was contained in 5 out of the top 10 liked tweets and in 3 out of the top 10 retweeted tweets. As political stance may play a role in the vaccine debate in the United States [9], it would be interesting for future studies to investigate its impact in addition to other factors.

This study showed that the overall valence of the tweets was positive. This was consistent with findings in prior research on tweets about vaccines in general [11-13] and about COVID-19 vaccines in particular, regardless of country [15,16]. The results showed that positive valence increased likes. This is in alignment with findings in prior research [22,23]. In comparison, the results showed no impact of valence on retweets. Past research revealed mixed findings regarding the effects of valence on retweets [11,25-27]. The explanation may rest in the complex cognitive sources underlying retweeting behavior. Compared with liking, retweeting is a more social behavior that may involve expected reactions from recipients about the content and/or the sender [26].

Regarding social media message features, account verification had the largest positive impact on likes and retweets among all factors, as reflected by *β* coefficients. This finding underscores the importance of account authentication in the popularity and virality of tweets in the face of massive amounts of information. Credible information is vital to reduce uncertainty in a crisis according to the uncertainty reduction theory [25,55]. However, it is notable that account authentication does not always mean content authentication. Accordingly, misinformation spread by verified accounts could pose greater challenges to vaccine campaigns. Vaccine campaigns could try to use and motivate different verified accounts, including institutional and individual accounts, to share credible information for wider reach and to prevent the spread of misinformation.

Furthermore, in alignment with the literature [32,33], the presence of a photo or video enhanced likes and retweets. The presence of a gif increased likes but did not affect retweets. In addition, consistent with the literature [23,34,35], the number of hashtags decreased likes and retweets. The number of external links decreased likes, but did not affect retweets. Inconsistent with the literature [23,25], the number of mentions facilitated likes, but did not affect retweets.

The results revealed that among the examined factors, more could impact likes than retweets. Eight topics predicted likes, whereas seven predicted retweets. Valence predicted likes but did not predict retweets. The presence of a gif, the number of mentions, and the number of external links predicted likes but not retweets. A comparison between like counts of the top 10 liked tweets and retweet counts of the top 10 retweeted tweets also suggested that a tweet was much more likely to be liked than to be retweeted. The number of likes for the highest liked tweet was more than five times the number of retweets for the highest retweeted tweet. These findings indicate more challenges to make a tweet viral than popular.

### Limitations

This study has several limitations. We used machine-based text mining to identify the underlying topics and valence in the vast amounts of tweets about COVID-19 vaccines. We then included the text-mined topics and valence, together with autoextracted information of social media message features in the regression models for prediction of the popularity and virality of tweets. Although this approach reduced manual coding, the results were mostly limited to autoidentified and autoextracted factors. Our manual reviews of sample tweets in each topic as well as the top 10 liked and retweeted tweets provided clues that politically polarized information could be intertwined with different topics. It would be interesting for future research to investigate how this may affect the popularity and virality of tweets. For instance, retweeting could derive from complex cognitive sources such as self-presentation [31] and identity communication [27]. A question arises whether consistency in the political stance between the sender and the recipients impact retweets.

Furthermore, the findings were limited to US-based public discourse about COVID-19 vaccines on Twitter. Social media platforms have played an important role in disseminating information and opinions during the COVID-19 pandemic [56]. It would be interesting for future research to compare Twitter with other social media platforms. For instance, the relative significance of examined factors in predicting popularity and virality may vary depending on the social media platform analyzed, as each has its own features.

Finally, the results revealed message-level drivers of the popularity and virality of tweets about COVID-19 vaccines. We included account verification as an independent variable in the regression models and the results showed that it had a positive impact on likes and retweets. However, we did not identify social bots in the massive amounts of tweets. It would be interesting for future studies to investigate the impact of social bots.

### Conclusions

This study suggests the public interest in and demand for information about vaccine development and people’s views, as well as vaccine efficacy and rollout during the COVID-19 pandemic. These topics, along with the use of media and verified accounts, enhance the popularity and virality of tweets. These issues could be addressed in vaccine campaigns to help the diffusion of content on Twitter.

## Abbreviations

BOW: bag of words
CDC: Centers for Disease Control and Prevention
LDA: latent Dirichlet allocation
mRNA: messenger RNA
WHO: World Health Organization
